# Supplementary material for: Geometry effects on protein mobility in a synapse
Source: Biophys J. 2025 Aug 8;124(18):3049–59. doi: 10.1016/j.bpj.2025.08.007 (PMC12709255; doi:10.1016/j.bpj.2025.08.007)
Supplement: Document S1. Figures S1–S5 and Tables S2 and S3 [file mmc1.pdf]

Biophysical Journal, Volume *124*

Supplemental information

Geometry effects on protein mobility in a synapse

Simon Dannenberg, Sofiia Reshetniak, Sarah Mohammadinejad, Silvio O. Rizzoli, and Stefan Klumpp

Supplementary Material: Geometry effects on protein mobility in a synapse

Simon Dannenberg,[1] Sofiia Reshetniak,[2] Sarah Mohammadinejad,[1] Silvio O. Rizzoli,[2] and Stefan Klumpp[1,*]

[1] *University of Göttingen, Institute for the Dynamics of Complex Systems, Friedrich-Hund-Platz 1, 37077 Göttingen, Germany*

[2] *University Medical Center Göttingen, Institute for Neuro- and Sensory Physiology, Humboldtallee 23, 37073 Göttingen, Germany*

[*] stefan.klumpp@phys.uni-goettingen.de

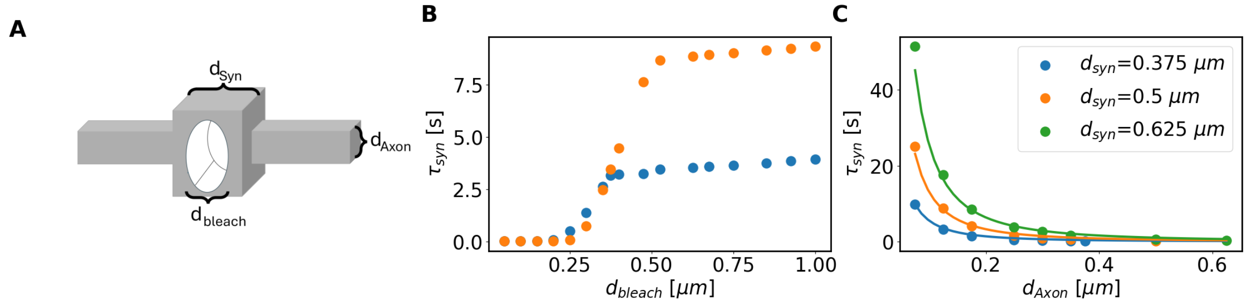

FIG. S1. Dependence of recovery times on geometric parameters. **A** Schematic of a simplified-geometry setup (box-shaped synapse) to investigate the geometry dependence of fluorescence recovery for simple instantaneous bleaching. **B** Dependence of the recovery time τ_{syn} in the synapse on the bleaching spot size for the scenarios with instantaneous bleaching and a box synapse of with $d_{syn} = 0.5$ μm. Note that the curve qualitatively changes as the entire synapse is bleached. **C** Relation between the recovery time τ_{syn} and the axon diameter d_{Axon} in the same idealized scenario for different synapse sizes in which d_{bleach} was chosen such that the entire synapse is bleached. The solid lines indicate a fit of the function $t_{syn} = \alpha \frac{V_{syn}}{A_{Axon}}$. Here, V_{syn} is the volume of the cubic synapse, and A_{axon} is the area of the axonal cross section. All three parameters $(d_{syn}, d_{Axon}, d_{bleach})$ were adjusted to fit the experimental setup as otherwise no fit with the experimental data from ref. 21 could have been obtained.

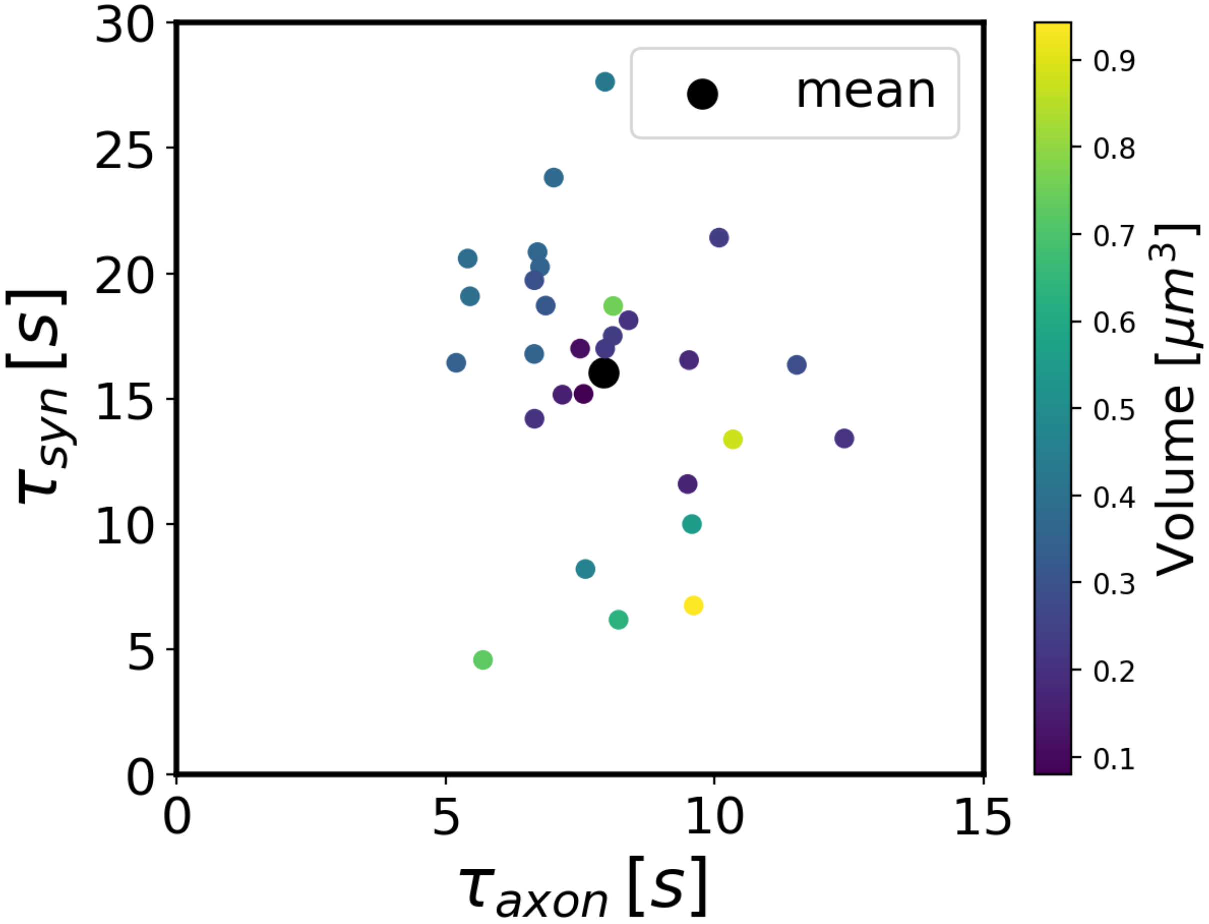

FIG. S2. Variability of the recovery times after photobleaching in different geometries: recovery times $\tau_{\rm axon}$ and $\tau_{\rm syn}$, in the axonal and synaptic regions, respectively. Each dot represents FRAP simulations in a different synaptic geometry. Simulations were done for pure diffusion with D = $0.1\,\mu m^2 s^{-1}$ and no binding to synaptic vesicles. The plot shows the same data as Figure 2C, but the color indicates the synapse volume rather than the long axis as in Fig. 2C).

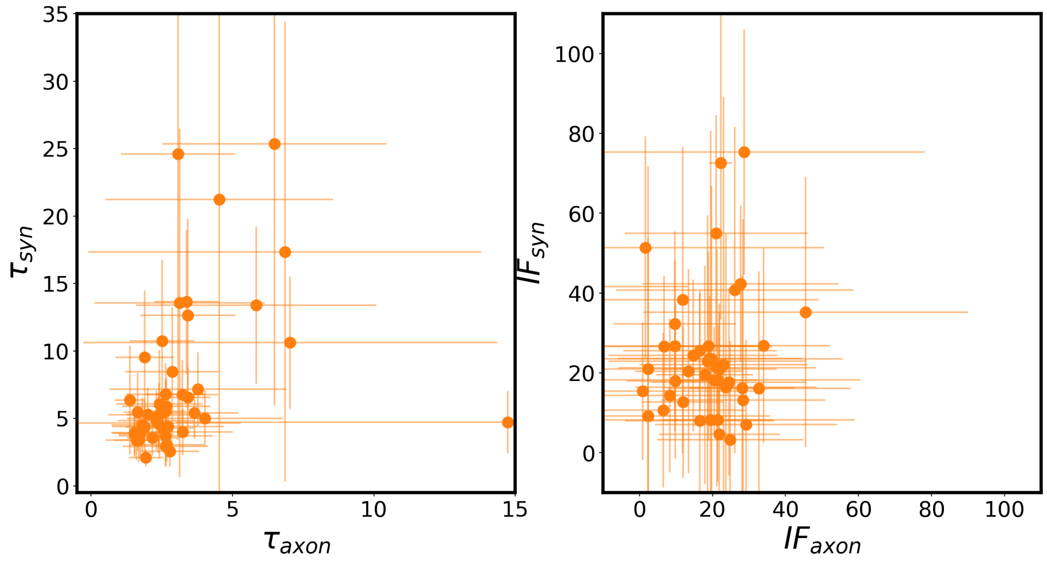

FIG. S3. Overview of FRAP results: Recovery times and immobile fractions in the synapse and the axon for 42 proteins (data from ref. 21).

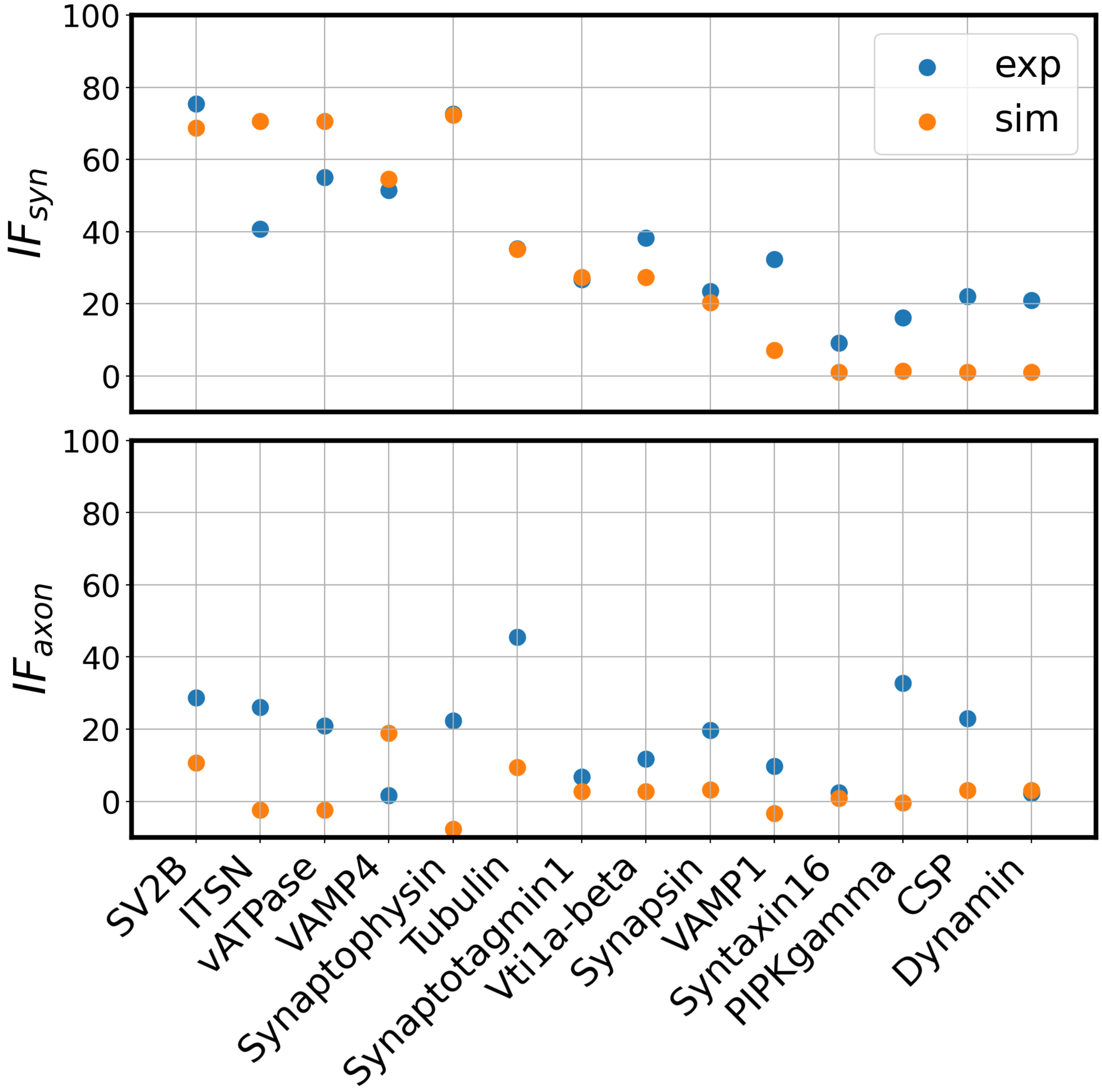

FIG. S4. Simulated immobile fractions (orange) obtained after matching simulations to experimental FRRAP data (blue). Note, that the experimental immobile fraction in the axon is not zero but rather centered around 20%. Similarly one can expect that this is effect is also present in IF_{syn} but masked by slower dynamics.

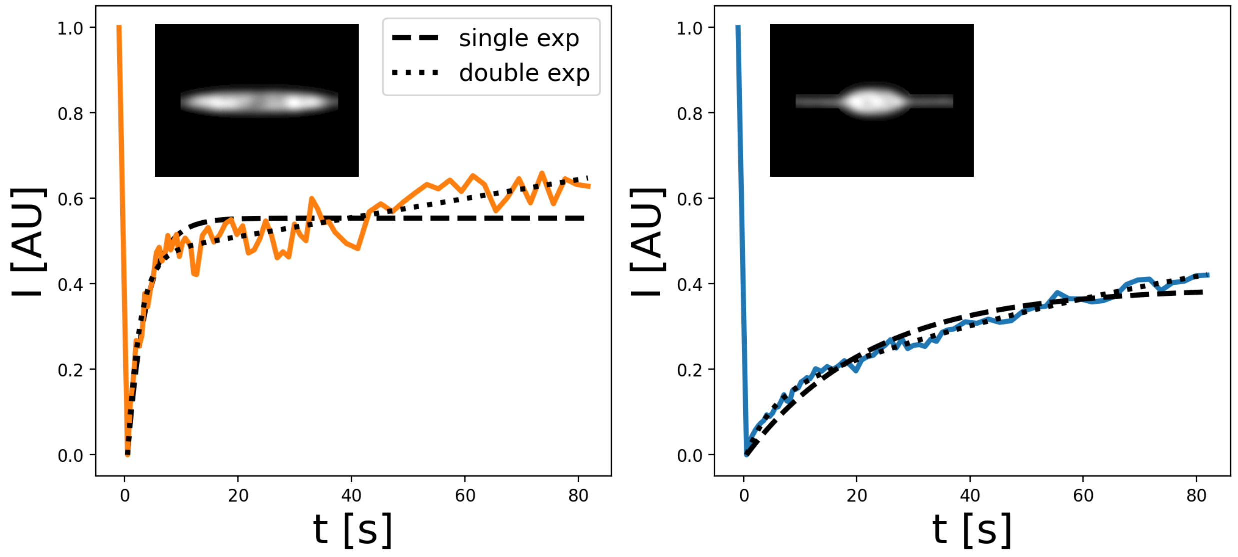

FIG. S5. Comparison of single and double-exponential fits to recovery curves. The recovery curves from Figure 2E (two individual simulations with the same diffusion coefficient) are fitted with a single exponential and with $I(t) = A_1(1 - \exp(-t/\tau_1)) + A_2(1 - \exp(-t/\tau_2))$. The insets show fluorescence intensity images taken directly after bleaching. In both cases, the double-exponential fit results in lower chi-square values. However, introducing four parameters can lead to overfitting and may wrongly attribute a second time scale to non-existent underlying effects such as binding. Prior examination of the microscopy images gives an indication of the presence of strong geometry effects: In the case of the larger synapse, a remnant of the bleaching spot is still visible, indicating geometric effects that lead to an apparent slower second time scale.

V [μm^3]	a [μm]	b [μm]	a_{mito} [μm]	#Vesicles	#Vacuoles	r_{vacu} [μm]
0.63	0.33	1.43	0.08	356	5	0.08
0.43	0.42	0.73	0.08	2064	5	0.10
0.46	0.28	1.47	0	788	10	0.10
0.38	0.41	0.64	0.08	1260	9	0.10
0.36	0.32	0.62	0.08	1028	4	0.10
0.87	0.49	0.84	0.12	2104	4	0.10
0.94	0.47	0.91	0.10	2036	9	0.18
0.55	0.29	1.26	0	1800	3	0.10
0.31	0.30	0.62	0	764	6	0.10
0.35	0.30	0.69	0	788	4	0.12
0.28	0.31	0.64	0	996	4	0.12
0.17	0.24	0.54	0	144	2	0.10
0.23	0.35	0.53	0.05	692	7	0.12
0.15	0.29	0.49	0	476	7	0.10
0.21	0.30	0.79	0	496	1	0.10
0.35	0.41	0.60	0	1508	8	0.10
0.39	0.36	0.67	0.08	740	5	0.10
0.34	0.32	0.79	0.10	544	7	0.10
0.29	0.33	0.67	0.10	848	5	0.10
0.76	0.40	1.01	0	2176	11	0.10
0.23	0.29	0.53	0.08	512	4	0.10
0.39	0.41	0.58	0.10	1008	10	0.10
0.21	0.31	0.40	0	932	2	0.08
0.08	0.27	0.38	0	344	3	0.08
0.11	0.33	0.33	0.08	380	3	0.10
0.21	0.38	0.38	0	1208	7	0.12
0.73	0.26	0.67	0	896	3	0.10
0.77	0.33	1.66	0.03	1612	11	0.10
0.17	0.33	0.47	0.10	548	5	0.08
0.23	0.27	0.87	0	992	2	0.10

TABLE S2. Overview of geometry parameters used to create synaptic structures in the simulations. Each row corresponds to one synaptic structure. V is the total volume of the synapse, a and b correspond to the short and the long axis of a rotational symmetric ellipsoid, respectively. a_{mito} the short axis of a rotational symmetric ellipsoid mimicking a mitochondrion. Its long axis $b_{mito} = 3a_{mito}$ and the values are chosen such that the occupied volume matches the reported values in EM measurements. #Vesicles and #Vacuoles are the numbers of vesicles and vacuoles in the simulation, respectively. While the radius of the SVs is always assumed to be 40 nm it can vary for the vacuoles and is chosen such that the total volume of vacuoles is preserved according to the EM measurements shown in the supplements of ref. [21].

Protein Name	Distance Deviation	D_{eff} deviation
CSP	[1.01, 1.03]	[0.8, 1.0]
Dynamin	[1.02, 1.03]	[0.8, 1.0]
ITSN	[1.16, 1.17]	[167.61, 125.7]
PIPKgamma	[1.1, 1.11]	[1.33, 0.67]
SV2B	[1.1, 1.15]	[0.6, 5.0]
Synapsin	[1.07, 1.15]	[0.67, 0.71]
Synaptophysin	[3.61, 5.78]	[0.5, 0.35]
Synaptotagmin1	[1.03, 1.14]	[1.0, 0.71]
Syntaxin16	[1.01, 1.06]	[1.0, 0.75]
Tubulin	[1.11, 1.14]	[0.67, 0.83]
VAMP1	[1.02, 1.02]	[0.7, 0.5]
VAMP4	[1.08, 1.2]	[1.67, 3.33]
vATPase	[1.63, 1.9]	[0.7, 2.0]
Vti1a-beta	[1.03, 1.06]	[1.0, 0.71]

TABLE S3. Overview of the fitting procedures uniqueness. We calculated the eucledian distances of the second and third best option as well and normalized it by the distance of the best fit. This gives us the normalized distance deviation $d_{dev} = d_{1st}/d_{2nd/3rd}$. Do gave an impression of the difference in dynamics between the different fit options we similarly calculated the differences in effective diffusion coefficients as they can serve as a single number mobility metric $= D_{eff_{dev}} = D_{eff_{1st}}/D_{eff_{2nd/3rd}}$. Values close to one in d_{dev} corresponded to equally good fits. Interestingly if the distance deviation is low in most cases there is also little difference in D_{eff} validating that the underlying dynamics are correctly described independent of the exact parameters.
